# Dr. John Tatham Thompson

**Published:** 1911-05-13

**Authors:** 


The death is announced of Dr. John Tatham Thompson, in his fifty-third year, of Cardiff, the youngest son of the late Silvanus Thompson, of York, and consulting ophthalmic surgeon at Cardiff Infirmary. He studied at Edinburgh University, where he graduated M.B. and C.M., having studied anatomy under Sir William Turner, and ophthalmic surgery under the late Professor Argyll Robertson. It was as an ophthalmic surgeon that Dr. Thompson gained his reputation. He was surgeon-oculist at the South Wales Institute for the Blind, at one time ophthalmic surgeon at the Western Dispensary, Edinburgh, and a vice-president of the Ophthalmological Society. He was author of "Miners' Nystagmus in the South Wales Collieries," "Operations for Staphyloma of the Cornea," "Cystic Detachment of the Retina," "Emphysema of the Conjunctiva," and "Influence of Early School Life on Eyesight." Dr. Thompson was a major in the R.A.M.C.

